# Super-enhancer acquisition drives oncogene expression in triple negative breast cancer

**DOI:** 10.1371/journal.pone.0235343

**Published:** 2020-06-25

**Authors:** Ryan Raisner, Russell Bainer, Peter M. Haverty, Kelli L. Benedetti, Karen E. Gascoigne

**Affiliations:** 1 Department of Discovery Oncology, Genentech, Inc., South San Francisco, California, United States of America; 2 Maze Therapeutics, South San Francisco, California, United States of America; 3 Department of Bioinformatics, Genentech, Inc., South San Francisco, California, United States of America; 4 Department of Cell and Tissue Biology, University of California, San Francisco, California, United States of America; Florida International University, UNITED STATES

## Abstract

Triple Negative Breast Cancer (TNBC) is a heterogeneous disease lacking known molecular drivers and effective targeted therapies. Cytotoxic chemotherapy remains the mainstay of treatment for TNBCs, which have significantly poorer survival rates compared to other breast cancer subtypes. In addition to changes within the coding genome, aberrant enhancer activity is a well-established contributor to tumorigenesis. Here we use H3K27Ac chromatin immunoprecipitation followed by sequencing (ChIP-Seq) to map the active cis-regulatory landscape in TNBC. We identify distinct disease subtypes associated with specific enhancer activity, and over 2,500 unique superenhancers acquired by tumor cells but absent from normal breast tissue. To identify potential actionable disease drivers, we probed the dependency on genes that associate with tumor-specific enhancers by CRISPR screening. In this way we identify a number of tumor-specific dependencies, including a previously uncharacterized dependency on the TGFβ pseudo-receptor BAMBI.

## Introduction

Triple Negative Breast Cancer (TNBC) is a heterogenous disease lacking clear molecular drivers. Transcriptional profiling has allowed stratification of the disease, primarily based on the similarity of tumors to a basal or luminal cell state [1]. While this distinction can help predict disease severity and outcome, it has yet to significantly impact treatment options. Previous TNBC profiling efforts focused on transcribed genes, evaluating gene expression, DNA mutations, and copy number changes. However, the non-coding regulatory elements that control gene expression are also critical to defining the tumor cell state, and remain relatively poorly explored in TNBC.

Gene-distal regulatory elements such as enhancers play an important role in the control of gene expression. Such elements are identified by their chromatin state, and the combination of histone modifications and chromatin binding proteins present. As such, technologies including chromatin immunoprecipitation followed by sequencing (ChIP-Seq) are required to interrogate these regions. In particular, acetylation of histone H3 at lysine 27 (H3K27Ac) is a well-established marker of active enhancers [2]. Genome-wide mapping and quantification of active enhancers has identified an asymmetry in their distribution, with a small subset of enhancers being significantly larger than the ‘typical’ [3]. These studies have defined a critical role for these ‘Super Enhancers’ (SE) in the regulation of gene expression in tumors, and in defining the transcriptional circuitry driving oncogenic growth [4]. In tumors, genes regulated by SEs are enriched for oncogenic transcription factors and factors controlling lineage specification and identify [5]. Such factors may not be commonly mutated or amplified in cancer, and as such are not readily detectible as oncogenic drivers by traditional evaluation of the protein-coding genome.

To better understand the role of acquired SE activity in TNBC we used genome wide H3K27Ac ChIP-Seq profiling to identify tumor-specific SEs, to which we assigned candidate cis-regulatory relationships on the basis of local gene expression. We then used CRISPR knockout screening to determine whether the genes associated with these tumor-specific SEs constitute vulnerabilities not previously identified by traditional profiling efforts.

## Results

### Defining the superenhancer landscape in TNBC

To better understand the gene-regulatory landscape in TNBC, we performed RNA-seq and ChIP-seq for histone H3K27Ac in a panel of 23 breast cancer cell lines and 10 primary tumors. Samples were defined as TNBC by an absence of Estrogen Receptor (ER), Progesterone Receptor (PR) or HER2 by Immunohistochemistry (IHC) or RNA expression. Three ER positive cell lines were also included for comparison. To enable the identification of enhancer elements specifically acquired by tumors but absent from normal breast tissue, we also profiled primary human mammary epithelial cells (HMECs) from two different donors, as well as hTERT immortalized HMECs, and vHMECS (variant HMECS which acquired immortalization spontaneously upon long term culture). To allow comparisons between tumors samples and distinct subtypes of normal breast cell, we included publicly available data sets from FACS sorted luminal-progenitor, luminal-mature, basal and stroma breast cells in subsequent analysis steps [6].

**Fig 1:**
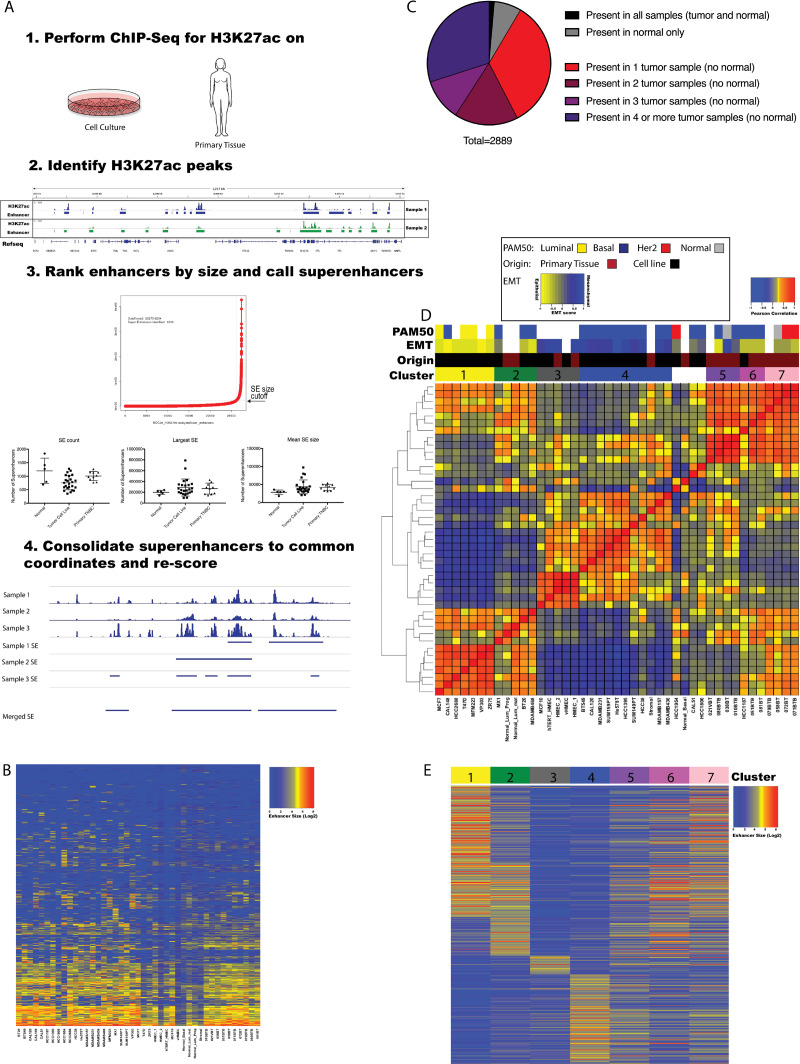
Defining the superenhancer landscape in TNBC. A) Workflow of ChIP-Seq data acquisition and scoring in primary and cell culture samples. Dot plots show the statistics on superenhancers size and count for samples derived from tumor cell lines, normal samples, and primary tumors. B) Heatmap of all samples input subtracted SE RPKMs scored against SE 20 kb fragment bins. C) Pie chart shows the distribution of SEs across all samples, that fall into the indicated categories. D) Hierarchical clustering heatmap of Pearson correlations of top 10% most variable SEs across all samples. E) Heatmap of cluster-specific SEs input subtracted RPKMs.

Enhancer and superenhancer (SE) elements were mapped and quantified by MACS and ROSE software using previously established parameters (Fig 1A and methods) [5]. An average of 23505 enhancers and 770 SEs were identified per tumor sample, and an average of 27764 enhancers and 980 SEs per non-tumor sample (S1 Table). These semi-redundant elements were consolidated across all samples into consensus maps (see methods). This merging resulted in a final composite map of 113,809 enhancers and 4,044 SEs for which each element was observed minimally in one sample. To standardize across samples each SE was split into 20 kb bins for downstream analysis (Fig 1B and S2 Table). Collectively these analyses revealed surprising variation in enhancers and SEs cross samples. Only 122 enhancers and no SEs were conserved across all samples in our data set. We identified 1,646 enhancers and 206 SE enhancers as present in normal but not tumor samples, and 23,946 enhancers and 2,643 SE present in at least one tumor sample but absent from normal samples. We also identified 7,581 enhancers and 979 SEs as present uniquely in a single tumor sample, indicating extensive variation and a net gain in the enhancer and SE repertoire in tumors (Figs 1B and 1C and S1A). To ask whether enhancer and SE patterns could be used to identify subgroups within our sample set, we used the top 10% most variable enhancers or SEs in our cell line samples to calculate the correlation across all samples and perform unsupervised hierarchical clustering (Figs 1D and S1B) [7,8]. In this way we identified seven prominent clusters of samples based on similarity of SE profile. We observed that using individual enhancers (not size restricted) the same broad clustering pattern was identified, however clusters were less well defined (S1B Fig). We therefore focused our subsequent analysis on SE data. We noted that Cluster 3 contained all HMEC samples. Clusters 5 and 6 and 7 were comprised of primary TNBC samples, and Clusters 1,2 and 4 contained a mix of cell line samples. Four of the samples showed no discernable similarly in SE profile to any other sample, and therefore were not assigned a cluster. To assess the impact of clustering breast cancer samples based on active cis-regulatory regions rather than steady-state gene expression, we compared unbiased clustering of the same samples based on SE profile and RNA-Seq profile (S1C Fig). While these analyses identified broadly similar clusters, resolution was significantly greater with SE correlation. In particular, while enhancer profiles of clusters 2 and 4 were anticorrelated (Pearson coefficient < 0), gene expression profiles between these groups were much more similar.

### TNBC SE profiles define unique tumor subsets

Analysis of the composition of the clusters defined by SE profile indicated unique characteristics of each. Assessment of an EMT gene expression signature across the profiled samples indicated that samples within Clusters 1 & 2 showed a more epithelial-like transcriptional profile, while cluster 4 samples expressed a more mesenchymal-like transcriptome (Fig 1D) [9]. Furthermore, Cluster 1 contained all hormone receptor (ER and AR) expressing cell lines samples. This was consistent with PAM50 classifications. Although primary tumor samples clustered separately from cell lines (likely due heterogeneity within primary sample cell composition as well as cell line adaptation to long term culture) there was still significant correlation between the sample types. Primary sample clusters 5 and 6 showed significant similarity to Cluster 4 (mesenchymal), while primary sample Cluster 7 showed high similarity to Clusters 1 and 2 (epithelial).

**Fig 2:**
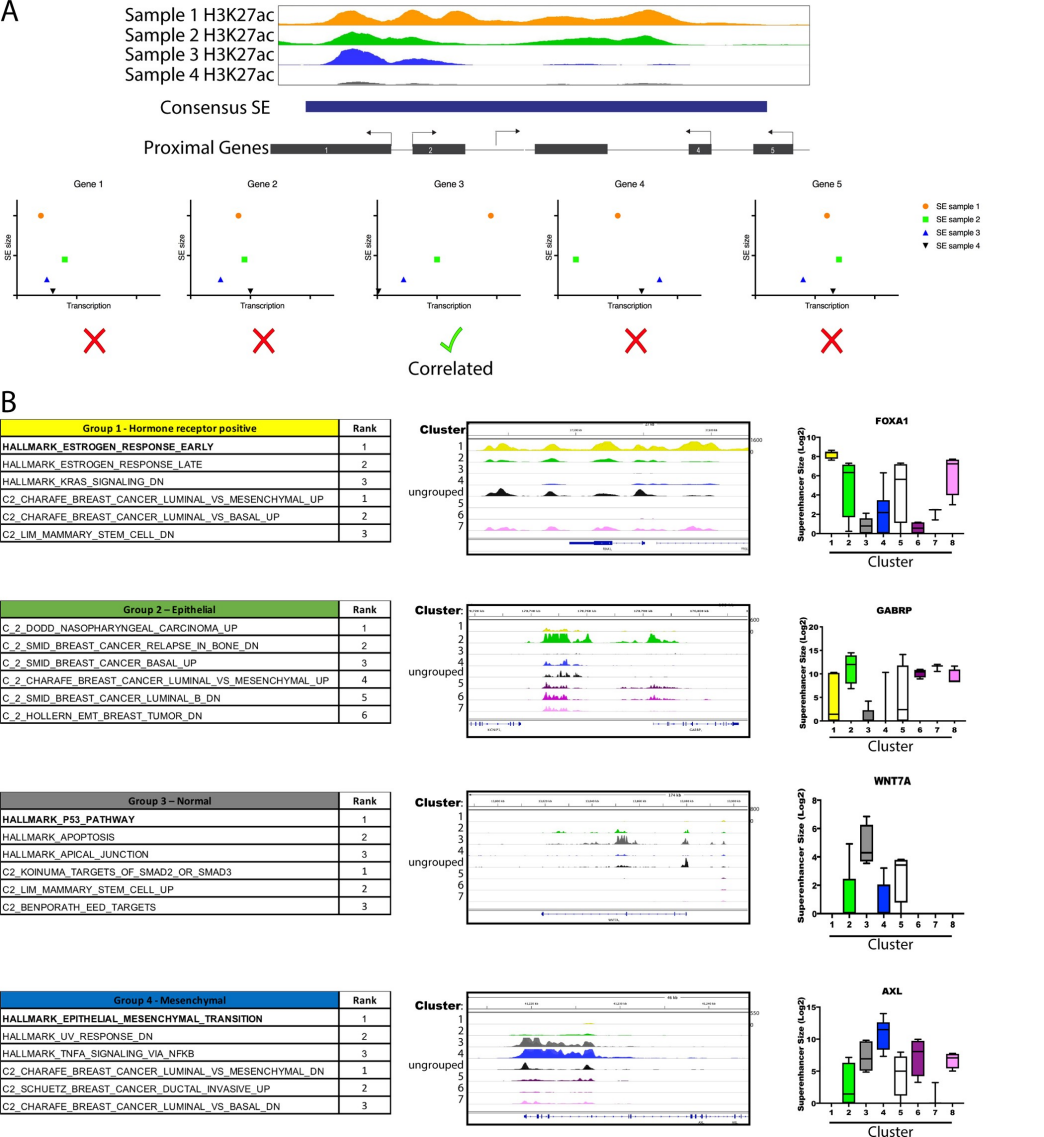
TNBC SE profiles define unique tumor subsets. A) Cartoon depiction of a fictional SE with overlapping and adjacent genes. Scatter plots show fictional example gene expression RPKMs vs. SE sizes, B) Gene Set Enrichment Analysis (GSEA) results for each subgroup. Left panel shows GSEA results for putative target genes from each Cluster-specific set of enhancers. Right panel shows example H3K27ac ChIP-Seq tracks and boxplots for superenhancers from each Cluster. Each track shown is an overlay of the tracks for all samples present in each group. Boxplots for each track example shows the total distribution of SE sizes for the samples in each Cluster. Mean, and interquartile range are shown; whiskers represent the minimum and maximum group values.

To further examine features driving clustering we identified the SEs whose activity best stratified Clusters 1, 2, 3 and 4 (Fig 1E and S3 Table). We also identified a large group of SEs as shared between Clusters 1 and 2. To better understand the gene networks controlled by these ‘cluster-defining’ enhancers, we sought to identify the genes most likely to be regulated by these elements, using a combination of proximity to the SE, as well as correlation between gene expression and enhancer size across the samples (Fig 2A and S4 Table) [7]. Briefly, expression of each gene within 10 Mb of a given SE was correlated to the presence or absence of the SE. Genes with a Pearson correlation co-efficient of 0.6 or great were then considered putative target genes of that SE. Interestingly, while the majority of correlating gene lay proximal to the associated SE, a subset lay much further away (up to 5Mb), suggesting the presence of long-range interactions (S4 Table and S1D Fig). Using this approach, we created a set of putative SE-regulated genes unique to each cluster (S5 Table). Gene Set Enrichment Analysis (GSEA) indicated that Cluster 1-defining enhancers predominately associate with genes involved with hormone receptor signaling. Similarly, both Cluster 2 and shared Clusters 1&2-defining enhancers regulated genes associated with the luminal phenotype, and Cluster 4 enhancers were associated with genes defining a mesenchymal / basal cell state (Fig 2B). Cluster 3 contained HMEC samples and consistent with this the SEs defining this cluster associated with genes defining normal cells.

### Tumor specific superenhancers drive oncogene expression

**Fig 3:**
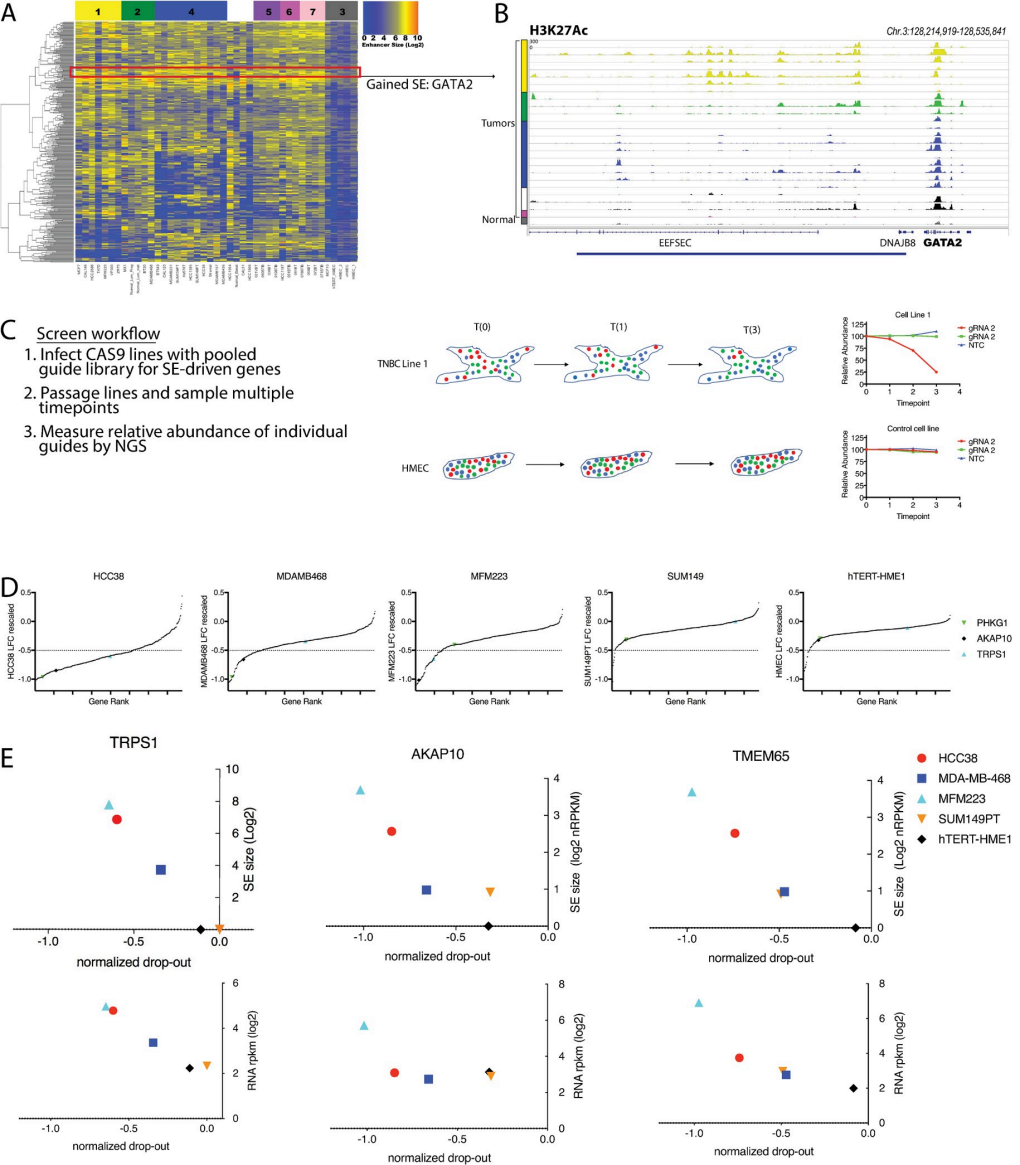
Tumor specific super-enhancers drive oncogene expression. A) Heatmap of tumor specific SEs across all samples as defined by scoring as a SE in at least 4 tumor cell lines and 1 TNBC primary tumor, and by not a SE in all normal samples. B) Example ChIP-Seq tracks for an acquired SE, putatively driving expression of GATA2. C) Diagram of screen workflow. D) CRISPR drop out screen results for tumor-specific SE associated genes in the indicated cell lines. E) Representative example screen hits. Top panel plots compare gene drop-out to SE size, bottom panel plots compare drop-out to RNA expression.

To define tumor-specific changes in the breast cis-regulatory landscape, we focused on SEs present only in tumor samples and not normal breast. We identified 2,643 SEs present in at least one tumor sample, but not found in normal samples. To remove potential artifacts present only in highly passaged cancer cell lines, we required tumor-specific SEs to be present in at least 4 TNBC cell lines, at least one primary TNBC sample, and absent from all HMEC samples (Fig 3A and 3B). We noted that in addition to tumor-specific enhancers identified in this way, a number of SEs were present in normal breast samples, but significantly enlarged in tumor samples. We included these regions in our analyses, defined as those enhancers where at least 2 times as many sequencing reads were counted in the region in tumor samples compared to normal, and using the additional criteria described above. Using these criteria, we identified 781 tumors-specific SEs. We hypothesized that these tumor-specific enhancers may confer a selective advantage during tumor development because they influence expression of genes critical for TNBC cell growth. In support of this notion we observed acquisition of tumor-specific SEs in proximity to known oncogenes such as EGFR and MYC, as well as a large number of genes whose role in TNBC has not yet been established (S6 Table).

To functionally validate the role of these SE-associated genes in tumor cell proliferation we designed a custom gRNA library targeting this gene set (S8 Table). We performed a series of CRISPR knockout screens in representative TNBC and normal breast cell lines, to determine whether acquired SEs could drive transcriptional activity of genes that represent TNBC-specific vulnerabilities (Fig 3C). Results of the screen identified 263 tumor-specific SE-associated genes were required for TNBC cell growth, as indicated by significant drop out from the infected population (Fig 3D and S7 Table). Genes which conferred selective drop-out in tumor lines compared to hTERT-HMEC were validated in arrayed format (S2 Fig). To assess the predictive power of SE acquisition for gene dependency, we evaluated the correlation between SE acquisition and drop out in screen data. This analysis revealed a number of vulnerabilities dependent on the presence of an associated tumor-specific enhancer, including known TNBC oncogenes and novel candidates (Fig 3E).

### BAMBI dependence is associated with superenhancer acquisition

**Fig 4:**
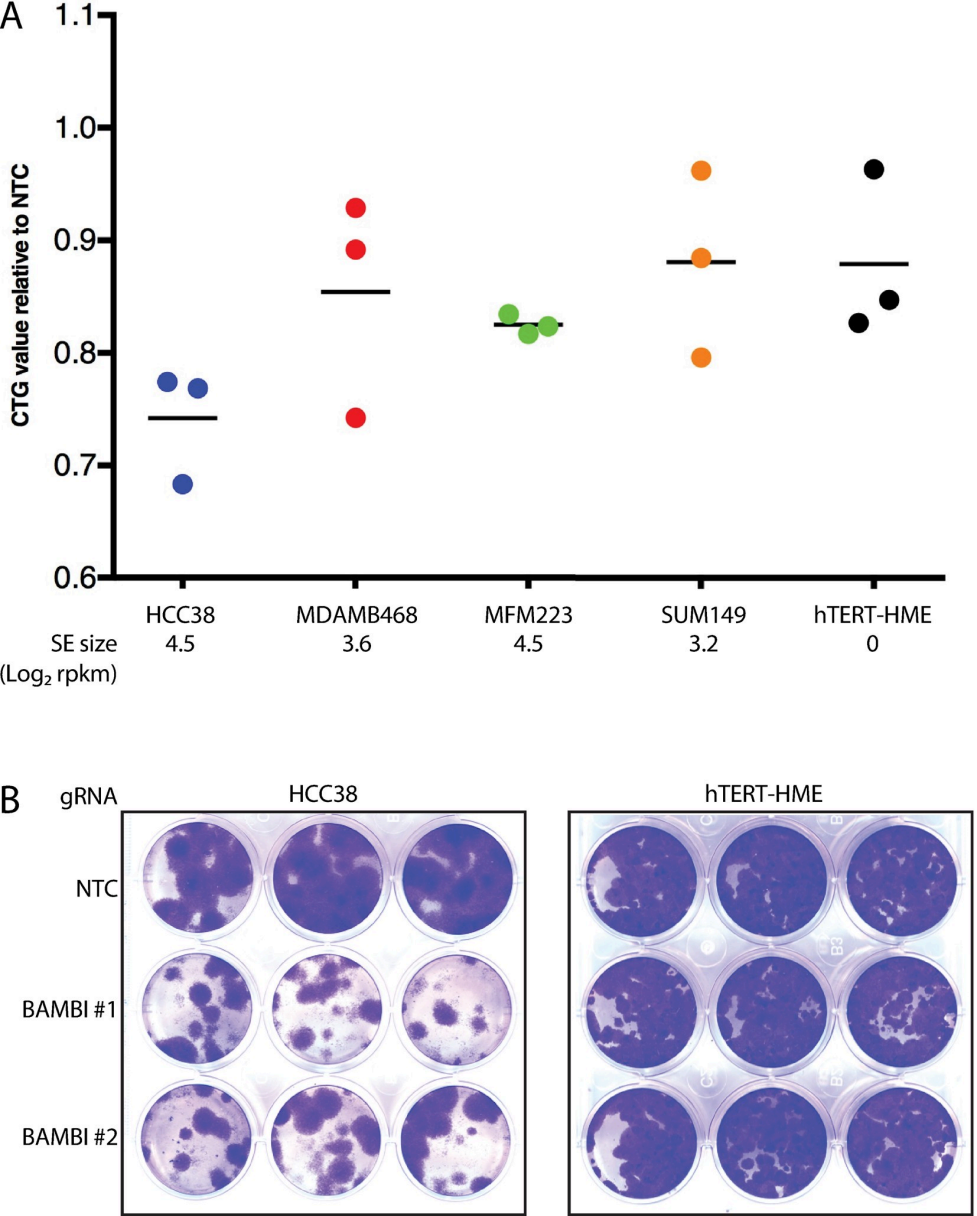
BAMBI dependence is associated with superenhancer acquisition. A) CellTiter-Glo® measurement of cell viability 4 days after BAMBI gRNA transfection. Each point corresponds to a different BAMBI targeted gRNAs, line indicates the mean. BAMBI SE size is indicated below each cell line. B) Colony formation assays 10 days after BAMBI gRNA transfection for the indicated cell lines.

**Fig 5:**
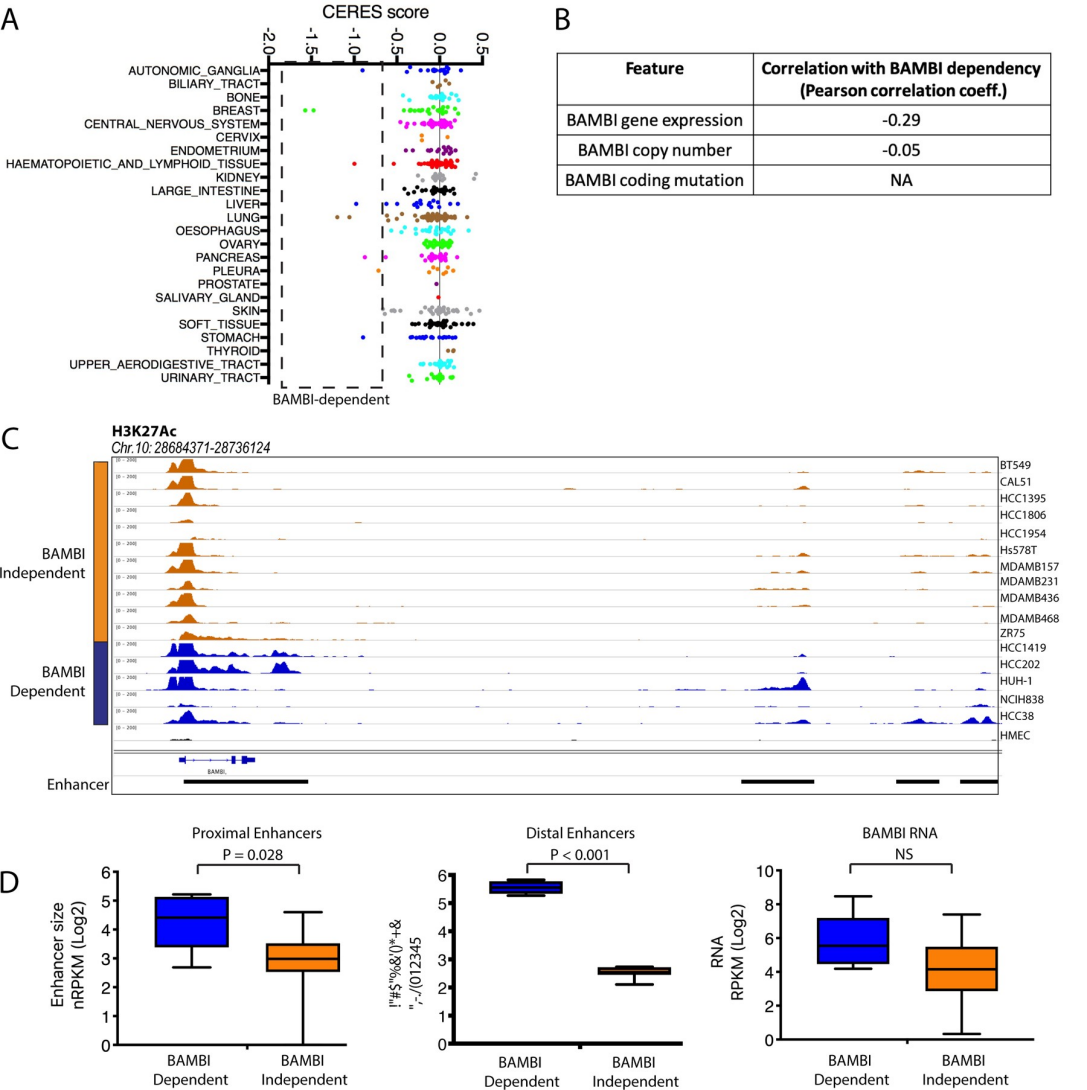
Fine-resolution analysis of BAMBI superenhancer landscape. A) Project Achilles data representing BAMBI knock out in 342 cancer cell lines. Drop-out is represented by CERES score. Box denotes all cell lines that have significant growth dependencies on BAMBI. B) Correlation between BAMBI dependency and the indicated genomic features in Project Achilles data. C) H3K27Ac ChIP-Seq tracks for BAMBI-independent TNBC lines, HCC38, and BAMBI dependent non-TNBC cell lines identified in project Achilles. High-variance enhancer regions are denoted below tracks. D) Left panel shows boxplot of summed BAMBI-proximal variant enhancer peaks annotated in C). Middle panel shows boxplots of summed BAMBI-distal variant enhancer peaks. Right panel shows boxplot of BAMBI RNA expression for BAMBI knock out sensitive and insensitive cell lines. Box indicates median, 25^th^ and 75^th^ percentiles, whiskers indicate min and max. * indicates significant difference P<0.001 as determined by Mann-Whitney U test.

We chose to further investigate one such candidate dependency, BAMBI, as a putative TNBC oncogene whose dependence correlates with SE association. BAMBI (BMP and Activin Membrane Bound Inhibitor) is a transmembrane glycoprotein most extensively studied for its role as an inhibitor of the TGFβ signaling pathway [10]. CRISPR knock out of the BAMBI gene was associated with significant growth inhibition in both long- and short-term growth assays in the TNBC cell line HCC38, while having no impact on growth of HMEC cells (Fig 4A and 4B). Dependency on BAMBI was confirmed by shRNA knockdown, and by suppression of the BAMBI TSS (Transcriptional Start Site) by CAS9 fused to the Krüppel-associated box (KRAB) and gRNAs targeting the BAMBI TSS (S3 Fig). Unexpectedly, the original SE associated with BAMBI based on BAMBI gene expression correlations across the samples did not correlate well with dependency (Figs 4A and S4A). However, additional enhancer and SE elements both proximal and distal to the BAMBI gene were identified which correlated well with BAMBI dependency. These were identified in HCC38 cells, as well as other BAMBI dependent cell lines identified in the Project Achilles cell line screening data set (Figs 5A–5D and S4B and S9 Table). Dependency in these lines did not correlate with BAMBI gene expression, copy number or mutation, suggesting an epigenetic predictor and a role for SE acquisition in BAMBI dependence in breast cancer.

**Fig 6:**
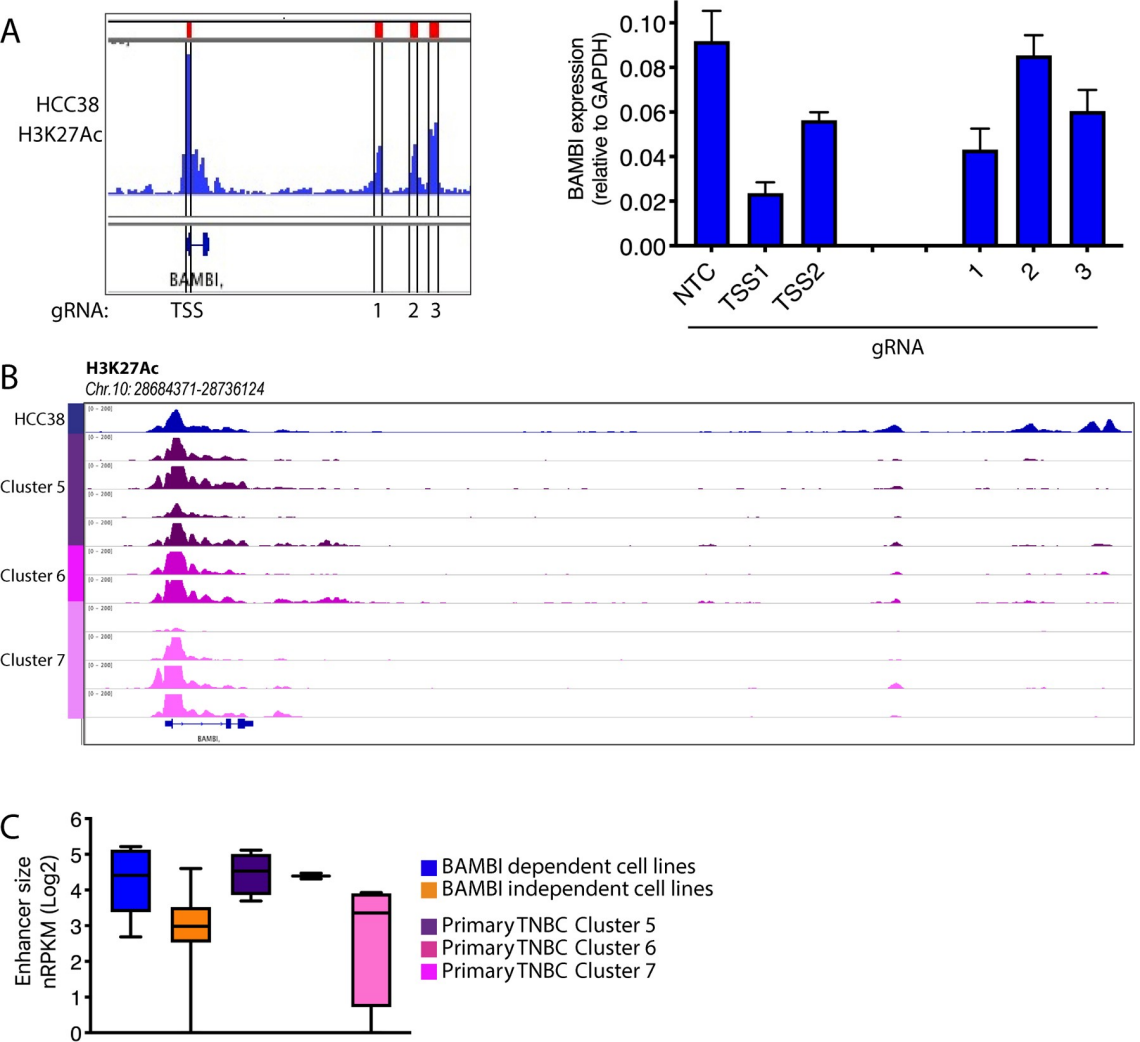
Enhancer regulation of BAMBI gene expression. A) Left panel shows H3K27Ac ChIP-Seq track of BAMBI-proximal region denoting enhancer peaks targeted by dCAS9-KRAB gRNAs. Right panel shows BAMBI expression by qPCR after cells were infected with the indicated gRNAs. B) Tracks show H3K27Ac ChIP-Seq signal of the BAMBI-proximal region in primary TNBC samples. C) Boxplot summarizing variant H3K27Ac peaks for the indicated groups. Box indicates median, 25^th^ and 75^th^ percentiles, whiskers indicate min and max.

To address the role of putative BAMBI enhancers in control of BAMBI gene expression, we used HCC38 cells expressing CAS9-KRAB and gRNAs targeted to individual enhancer peaks, as well as the BAMBI TSS, to silence individual chromatin regions (Fig 6A). As expected, gRNAs targeting the BAMBI TSS were able to repress BAMBI expression. Additionally, enhancer-targeted gRNAs gave a range of repressive activity on BAMBI transcription, allowing identification of enhancer peaks with the greatest influence on gene expression (Fig 6A). Due to adaptation to long-term 2D culture, well established differences exist between cell lines and primary tumor samples. However, BAMBI-proximal enhancers that correlated with dependency in cell lines were also identified in TNBC primary samples, suggesting such elements are active in primary disease (Fig 6B and 6C). We also observed H3K4me3 peaks at a number of these variable enhancer regions in primary TNBC samples, suggesting that expression of non-coding transcripts in this active cis-regulatory region may play a role in BAMBI regulation (S5 Fig).

## Discussion

The dysregulation of gene expression in cancer is well established, and cannot be adequately explained by genomic alterations such as coding gene mutation and copy number changes. Instead, such widespread changes are attributed to the extensive transcriptional rewiring that occurs in cancer cells, including the utilization of different transcription factor repertoires, and the activation of alternative gene-regulatory elements including enhancers [11–14]. A deeper understanding of the epigenetic and transcriptional landscape of tumor and normal cells has helped shed light on the mechanisms behind some of these changes, however, much remains to be understood about how they occur and their role in tumorigenesis.

As a disease which lacks molecular drivers and targeted therapy options, TNBC is an area where a deeper understanding of the epigenetic changes that occur during tumorigenesis may be particularly impactful. With this in mind, here we describe the active cis-regulatory landscape of TNBC. Based on the epigenetic and transcriptional profiling of 10 primary tumors, normal HMECs from multiple donors, and 23 TNBC cell lines, we document the changes that occur in the enhancer landscape in tumor cells compared to their normal counterparts. Unsurprisingly, significant differences were observed between primary and cell line populations, and the use of cell lines in this analysis is caveated by their undoubted divergence from primary tumors under the selection pressure of long-term 2D growth. Importantly however, many commonalities were also identified in the cis-regulatory landscapes, and cell line inclusion in this study allowed follow up and functional validation not possible in primary tissue.

Previous work has uncovered a surprising asymmetry in the distribution of enhancer size genome wide, with a subset of enhancers in every cell being significantly larger than the average. While the exact size of these SE elements varies considerably between cell types, the asymmetric distribution is constant, and a set of SEs can be defined in every sample, as those falling beyond the inflection point of a curve ranking all enhancers in a given sample by size [15]. Biological characterization of the function of these elements and the genes they regulate has uncovered a bias towards the regulation of lineage-defining transcription factor networks, and in the context of cancer, in the regulation of oncogenic transcription factors [4,5]. Indeed, there is precedent in a number of indications for the mapping and quantification of SE size to be used to identify oncogenic transcriptional circuitry of importance for tumorigenesis [3,7,8,16–18]. Here we apply this same approach to TNBC. While the cis-regulatory landscape of any cell is undoubtedly vast and complex, in this study we chose to focus only on SE elements, and test the hypothesis that identification of the largest enhancers in primary and cell line TNBC samples will help identify critical oncogenes and transcriptional networks in the disease.

Using histone-H3K27Ac ChIP-Seq and analysis using MACS and ROSE software, we mapped and quantified enhancers genome wide in TNBC samples. Using established ROSE parameters consistent with other studies (see methods), we identified the subset of enhancers in each sample considered superenhancers. Consistent with prior studies, we did not exclude TSS elements from our ROSE derived SE calls, due to the significant overlap between SE regions and TSS and gene bodies. We noted a large number of SE present in at least one tumor sample but absent from normal breast samples, indicated a gain in SE activity in tumors. Using unsupervised hierarchical clustering we compared SE landscapes across TNBC and normal breast samples, and were able to define subgroups of samples with more similar profiles. Most prominently, clustering samples using the most variable SEs in the data set stratified samples into epithelial and mesenchymal populations. Strikingly, although these broad groupings could also be observed in transcriptome data, the separation between the groups was significantly greater in the SE data set. In particular, SE profiles for epithelial vs mesenchymal samples were anti-correlated in the SE data, while positively correlated in the transcriptomic data set. Similarly, while SE profiling was able to identify 3 different subgroups of primary TNBC samples, these were indistinguishable based on their transcriptomes. Finally, several cell line samples were un-clusterable using SE data, having enhancer profiles so unique they did not significantly correlate with any other samples, suggesting a highly unique gene-regulatory landscape in these cells. In contrast, these cell lines did not stand out in transcriptome data, having similar gene expression profiles to other more mesenchymal samples. Taken together, these data suggest that information on SE activity in tumor cells provides additional insight into the cancer cell state, beyond that of steady state gene expression. Furthermore, the observation that cells with similar transcriptomes can have strikingly different enhancer profiles indicates a previously unappreciated diversity in the regulatory landscape of TNBC. While outside the scope of this study, profiling the variations in transcription factor occupancy at these sites may afford a deeper understanding of the factors dictating this diversity, and the opportunity to target these unique states.

Comparison of TNBC and normal breast SE profiles identified a large number of SEs present in tumor cells but absent from normal tissue, indicating that tumor-specific enhancer acquisition is common. To understand the link between these alterations in the SE landscape and gene expression changes in TNBC, we used an informatics approach to identify putative SE-regulated genes, by correlating SE proximity and gene expression across samples to predict genes most likely to be regulated by a given SE. This approach is caveated by the long-range interactions possible between enhancers and promoters, and the likely possibility that within a cell population an enhancer may regulate several genes, and vis versa, a gene may be regulated by several enhancers. To accommodate this, gene expression correlations within 10 MB of each SE were considered, as were multiple genes correlating with an individual SE. Previous studies have shown this approach to cross-validate well with Chromatin Conformation Capture assay data [7]. Analysis of gene sets associated with SE defined groups of TNBC samples again indicated a stratification based on epithelial vs mesenchymal cell state, as well as hormone receptor expression, and correlated broadly with PAM50 classification. Consistent with previous observations, genes associated with tumor-specific SEs included known oncogenes and lineage-defining transcription factors, as well as genes not previously associated with TNBC.

Previous efforts to characterize the epigenetic landscape in cancer have been primary descriptive. Here we expand to interrogate the functional role of these putative tumor-specific SE regulated genes in TNBC growth and survival. Using CRISPR dropout screening we test the hypothesis that acquisition of SE regulation during tumorigenesis may predict a dependence on the associated gene. Consistent with this hypothesis, we identified 263 tumor-specific SE regulated genes that were required for TNBC proliferation but not for growth of normal breast cells. We chose to follow up on one such tumor-specific dependency which represents a potential targetable vulnerability. We identified the TGFβ pseudo-receptor BAMBI as a SE associated gene whose loss inhibited cell growth in HCC38 TNBC cells, but not in normal HMEC cells. Analysis of Project Achilles cell line screening data identified a number of additional cell lines also dependent on BAMBI for growth. This dependency could not be predicted by known features such as expression, mutational or copy number status. Upon detailed analysis of the enhancer landscape we identified variable enhancer regions where enhancer presence correlated well with BAMBI gene dependency. These enhancers were located in both BAMBI proximal and distal regions. We hypothesize that while these enhancers may not directly control BAMBI expression, they may reflect or control a cell state that dictates a dependency on BAMBI. Importantly, these variant enhancers were also present in primary tumor samples, predicting BAMBI dependency in TNBC patients.

In conclusion, this study provides the first comprehensive, integrated map of the gene expression and cis-regulatory landscape of TNBC. Analyses of these data indicate that when combined with traditional cancer genomic and expression data, gene-regulatory information can improve our understanding of the cancer cell state, in particular it’s diversity. It is interesting to speculate that the great diversity of enhancer usage seen in this study may represent unique transcription factor circuitries active in difference tumors. Although leading to common gene expression outputs, these unique circuitries may represent targetable vulnerabilities in subsets of patients. The integrated approach taken here to uncover this diversity may provide additional information for both biomarker and drug target discovery efforts in TNBC, and could be applied to other cancer types with a paucity of known molecular drivers.

## Materials and methods

### Cell culture and proliferation assays

All cancer cell lines in this study were obtained from ATCC, authenticated by STR analysis and confirmed mycoplasma negative by PCR test. All cancer cell lines were cultured in RPMI media supplemented with 10% fetal bovine serum and 2 mM glutamine. hTERT-HME cells were obtained from ATCC, authenticated by STR analysis, confirmed mycoplasma negative by PCR test and grow in MEBM media supplemented with growth factors (Lonza cat# 3151 and #4136). Primary HMEC cells (ThermoFisher cat# A10565) were grown in HuMEC Ready media (ThermoFisher cat# 12752–010). Puromycin concentration for all viral infected cultures was maintained throughout growth at a concentration of 2 ug/mL. CAS9 stable lines were selected for and maintained with Blasticidin at a final concentration of 2.5 ug/mL. Cell proliferation was evaluated in a 384-well format using CellTiter-Glo® reagent (Promega) according to manufacturer’s instructions, or by assessment of confluence using the Incucyte Zoom platform in a 12 well plate format, with brightfield images acquired every 5 hours.

### Primary samples

Frozen triple negative breast cancer tumor samples were acquired from Cureline (South San Francisco). Samples were homogenized via mechanical disruption before processing for RNA-Seq and ChiP-seq as described below.

### Cell line generation

CAS9 expressing cell lines were generated by infection with pLenti 6.3 CAS9 virus, followed by selection with Blasticidin. Pooled cultures were maintained in the presence of Blasticidin and infected with sgRNA virus targeting PLK1, or non-targeting controls (NTC) to test for CAS9 activity. All cell lines had >80% killing after infection with PLK1 targeting gRNAs, as assessed by CTG assay.

### Clonogenic assays

Cells were plated at densities of 500, 1,000, or 2,000 cells per well and allowed to adhere overnight in RPMI 10% FBS media. Wells were infected with normalized titers of guide virus with a target MOI of 1. After 3 days post-infection, fresh media including puromycin 1 ug/mL was added to each well. Media was changed every 3–4 days until control wells reached confluence. Colonies were visualized by staining with 0.5% Crystal violet for 30 minutes at room temperature.

### Pooled CRISPR screening

Pooled CRISPR screening was performed in non-clonal CAS9 expressing cell lines as described above and according to [19], using 8 sgRNAs per gene, with a total of 3875 sgRNAs in the library (S8 Table). A MOI of 0.3 was targeted in each cell lines. The impact on growth of each gene in the library was assessed by measuring the change in abundance of each gene’s 8 sgRNA guides from the reference timepoint (t = 0) to a late timepoint (t = 3). The top 3 guides per gene were used to calculate the log fold-change (LFC) for each gene for each cell line. In order to cross-compare data across different cell lines with varying CAS9 activity, LFC data was normalized to positive (essential genes) and negative (NTC) control genes so that a value of -1.0 corresponded to the average value of our positive controls, and 0.0 corresponded to the average of our NTCs. All values at or below -0.5 were considered to be putative ‘hits’.

### Gene expression analysis

RNA was purified from cells using the RNeasy kit (Qiagen) according to manufacturer’s instructions. Quantitative RT-PCR was performed using Taqman assay (ThermoFisher Scientific, Inc.) on ABI QuantStudio 7 Flex real-time PCR system. For whole transcriptome RNA-sequencing RNA libraries were made using TruSeq RNA Sample Preparation Kit v2 (Illumina). Size of the libraries was confirmed using Fragment Analyzer (Advanced Analytical Technologies) and their concentration was determined by qPCR-based method using Library quantification kit (KAPA). The libraries were multiplexed and then sequenced on Illumina HiSeq2500 (Illumina) to generate 30M of single end 50 base pair reads. Gene set enrichment analysis (GSEA) was performed using Broad Institute software (http://software.broadinstitute.org/gsea/index.jsp).

### Chromatin immuno-precipitation (ChIP)

Following treatment 1 million cells or 500 mg of primary tumor were crosslinked in 1% formaldehyde for 15 minutes, then quenched with 125 mM glycine for 5 minutes. Primary tumors were mechanically dissociated, then all cells were lysed and chromatin sheared by sonication to an average length of 300–500 bp. Genomic DNA (Input) was prepared by treating aliquots of chromatin with RNase, proteinase K and heat for de-crosslinking, followed by ethanol precipitation. Pellets were resuspended and the resulting DNA was quantified on a NanoDrop spectrophotometer. Extrapolation to the original chromatin volume allowed quantitation of the total chromatin yield. 30 μg of chromatin was precleared with protein A agarose beads (Invitrogen). Immunoprecipitation was then carried out using the following antibodies: anti-H3K27Ac (Active Motif catalog # 39133, lot #31814008, 4 ug per ChIP), anti-H3K4me3 (Active Motif catalog # 39159, lot # 15617005, 3 ul used per ChIP).

ChIP-qPCR, immunoprecipitated complexes were washed sequentially with low to high-salt wash buffers, followed by a wash in TE, followed by elution from beads with TE + SDS 1%. Eluates were treated with 10 μg of RNaseA for 30 minutes at 37°C, followed by 20 μg of Proteinase K for 30 minutes at 55°C. Crosslinks were reversed by incubation overnight at 65°C, and ChIP DNA was purified using QIAQuick PCR purification columns (QIAGEN).

ChIP and Input DNAs were prepared for amplification by converting overhangs into phosphorylated blunt ends and adding an adenine to the 3’ ends. Illumina genomic adapters were ligated and the sample was size-fractionated (200–300 bp) on an agarose gel. After a final PCR amplification step (15 cycles), the resulting DNA libraries were quantified and sequenced on Illumina NextSeq 500, producing 75 nt reads, single ended reads. (For sequencing QC metrics see S1 Table).

### CRISPRi cell line generation and assay

Repression of enhancer activity was achieved by stable expression of dCAS9-KRAB in HCC38 cells and then lentiviral infection of pLKO-based plasmids containing a gRNA targeting the BAMBI transcriptional start site (TSS-1) (5′-GCGTCCCTAGAGTCGAGCG-3′), (TSS-2) (5′-AGCAACTTGTCGCGACCTG-3′), extragenic regions of the BAMBI enhancer (enh_1) (5’-CCTATATGTGAATCCACCT-3’), (enh_2) (5’-GTAATCCCAACTACTCCGG-3’), (enh_3) (5’-AGTCAGTATACCAACACTG-3’), (enh_4) (5’-GAACCTGGACATCCTCCAC-3’), (enh_5) (5’-AGACCGGGTTTCAGCACGT-3’), (enh_6) (5’-ATGTAACACATACCCACTG-3’), (enh_7) (5’-CCCCACGTAGCATCACCCA-3’), (enh_8) (5’-GTCTAATGTGTGATAACTG-3’), or a negative control gene desert region (5′-TCCCCCTCAGCCGTATT-3′). Cells were processed for RT-qPCR analysis after 5 days post-infection.

### shRNA line construction and conditions

HCC38 cells were infected with virus containing doxocycline-inducible pZIP-TRE3G shRNA constructs (Transomics) for 3 different BAMBI-targeting sequences (BAMBI sh1 5’-TGCTGTTGACAGTGAGCGAAAGCAGACCTCAGCAACGATATAGTGAAGCCACAGATGTATATCGTTGCTGAGGTCTGCTTGTGCCTACTGCCTCGGA-3’), (BAMBI sh2 5’-TGCTGTTGACAGTGAGCGACTGAGGATGCTTCGAAGTGAATAGTGAAGCCACAGATGTATTCACTTCGAAGCATCCTCAGGTGCCTACTGCCTCGGA-3’), (BAMBI sh3 5’-TGCTGTTGACAGTGAGCGAGGCACGAGAACTGCTGTCTGATAGTGAAGCCACAGATGTATCAGACAGCAGTTCTCGTGCCCTGCCTACTGCCTCGGA-3’), or an NTC sequence. Inducible shRNA expression was confirmed by GFP expression. For clonogenic, incucyte, and RNA samples, shRNA cell lines were induced for 2 days with 1 ug/mL doxycycline, following which cells were trypsinized, counted, and re-seeded for cell growth assays and RT-qPCR in the presence of doxycycline.

### Statistical analysis

#### ChIP-Seq data analysis

Reads were aligned to the human genome (GRCh38) using the GSNAP algorithm, (http://research-pub.gene.com/gmap/ version 2013-10-10) with the following settings: “-M 2 -n 10 -B 2 -i 1—pairmax-dna = 1000—terminal-threshold = 1000—gmap-mode = none—clip-overlap”. Fragment length was determined by the strand cross-correlation method. Reads were extended to this fragment length before coverage was calculated at a per-nucleotide level using uniquely mapping reads.

#### Enhancer and superenhancers peak identification and scoring

H3K27ac ChIP peaks were identified by the MACS version 2 software package [20] in conjunction with paired input DNA samples with the callpeak function using default settings, genome set to ‘hs’, and peak calling set to—broad. Enhancer and Superenhancer peaks were identified with the ROSE version 1 software package [5,15] using the MACS output files. ROSE software was executed with default parameters of 12.5 kb stitching distance, and TSS exclusion size set to 0, with the genome set to hg19. MACs and ROSE output statistics are described in S1 Table. Individual enhancers coordinates were derived from initial MACS output tables and filtered based on maximum p-value = 0.05 and empirically determined peak sizes relative to paired input samples. Superenhancer consolidation across samples was accomplished using Bedtools version 2.2 software -merge function with a minimum overlap distance of 5000 bp between enhancers [21]. Enhancer consolidation was done with Bedtools -merge function with a minimum overlap of 10 bp between enhancers. After merging enhancers into a consensus map, enhancers that overlap TSS coordinates were removed using the Bedtools -subtract function. Enhancers and superenhancer scoring for all samples against their respective consensus enhancer / SE map was performed by scoring each sample’s IP and input alignment (.bam) files using the bedtools -multicov function to determine the total reads for each element interval per sample. Raw coverage values were then adjusted for read-depth, and the adjusted coverage values for input were subtracted from their respective paired ChIP sample to give a final score for each enhancer coordinate for each sample. The absolute value for a SE cutoff per sample is variable due to the ROSE software and distribution of enhancer sizes across samples, therefore a value of Log2(RPKM+1) = > 6 was adopted as the SE cutoff threshold for sample scores according to the consensus SE map. This score is inclusive to all of the originally identified SE from all samples. All statistical comparisons of superenhancers between groups of cell lines were performed with 2-tailed unpaired T-tests.

#### Superenhancer clustering

The top 10% most variable superenhancers were measured by calculating the variance of SE sizes for all cell line samples. Hierarchical clustering and heatmaps for SE data was done on the high variance SE with the ggplot (https://ggplot2.tidyverse.org) and heatmap.3 (https://gist.github.com/nachocab/3853004) R packages, using the hclust average linkage algorithm for calculating distances. Optimal cluster number was determined empirically by testing various cluster sizes with the “Elbow Method”, “Sillouette Method”, and “Gap statistic”. Based on the output of those tests and prior knowledge of our samples’ biological state, a cluster number of 10 was chosen. Group assignments were done for clusters with 3 or more samples. SEs were assigned to groups 1–4 according to the criteria that the mean group value minus the standard deviation did not overlap with the mean value of any of the other groups. In the case of the SEs shared between groups 1 and 2, the group means minus standard deviations populations were overlapping with one another, but were distinct from groups 3 and 4.

#### Gene to superenhancer mapping

To measure the correlation between genes and superenhancers, for each superenhancer the Pearson correlation between superenhancer size (log2) of each cell line was calculated to the gene RPKM value (log2) for all genes within 10 Mb of the enhancer coordinates. Genes which have a Pearson correlation of 0.6 or greater were considered to be associated with a given superenhancer. For each superenhancer, a minimum of two genes with the highest correlations were assigned, regardless of a minimum value. In cases where 3 or more genes were highly correlated to a SE, all genes above a correlation of 0.6 were deemed to be associated with the SE.

#### Achilles data analysis

Dependency data was extracted from the Achilles cell line screening project using the 2018 Q4 public release of data (https://depmap.org/portal/achilles/). Cell lines with a CERES drop-out score less than or equal to -0.5 were categorized a sensitive to BAMBI knockout.

#### Defining BAMBI-distal SE signatures

Foe BAMBI dependent and independent cell lines correlation matrices were created to identify superenhancers that were most positively and negatively correlated with BAMBI dependency. We performed unsupervised hierarchical clustering of the log2 ChIP data for both the top 600 positively- and top 600 anti-correlated superenhancers. The signature size of 600 enhancers was determined empirically to give an optimal p-value for distinguishing between BAMBI-sensitive and BAMBI-insensitive samples.

#### Accession numbers

The accession numbers for the RNA-Seq and ChIP-Seq data reported here are ENA: PRJEB33558

## Supporting information

S1 Fig Enhancer and RNA correlations across samples. A) Pie chart shows the distribution of enhancers that fall into the indicated categories. B) Hierarchical clustering heatmap of Pearson correlations of top 10% most variable enhancers across all samples. C) Hierarchical clustering heatmap of Pearson correlations using RNA-seq values of top 10% most variable genes. D) Scatterplot shows the distance of the best correlating genes to their associated SE.
(TIF)

S2 Fig Primary validation of dropout screen results. Plots show arrayed validation of primary screen hits for all CAS9 lines. Whiskers denote max/min values. Middle bar indicates mean value.
(TIF)

S3 Fig Orthologous validation of the BAMBI phenotype. A) RT-qPCR quantification of BAMBI TSS-targeting dCAS9-KRAB guides in HCC38 cells. B) Clonogenic assays for BAMBI-TSS and “gene desert” targeting gRNAs in dCAS9-KRAB HCC38 cells. C) Quantification of clonogenic assay wells. D) RT-qPCR quantification of shRNA knockdown of BAMBI upon doxycycline induction for 3 independent shRNA constructs, and a non-targeting control shRNA at 7 days post-induction. E) Clonogenic assays of HCC38 cells after doxycycline induction of the indicated shRNAs. F) Incucyte growth assays of HCC38 cells after doxycycline induction of the indicated shRNAs.
(TIF)

S4 Fig SE acquisition predicts BAMBI dependence. A) H3K27ac ChIP-Seq tracks of the BAMBI-adjacent region for the indicated cell lines. BAMBI associated superenhancers are indicated below the tracks. B) Heatmap showing top correlating SEs with BAMBI dependence.
(TIF)

S5 Fig Dynamic H3K4me3 at the BAMBI in primary TNBC. Tracks show H3K4me3 signal at the BAMBI locus in primary TNBC samples.
(TIF)

S1 Table ChIP-Seq QC statistics and Rose output statistics. (XLSX)

S2 Table Consensus enhancer and superenhancer coordinates. (XLSX)

S3 Table Cluster specific superenhancers. (XLSX)

S4 Table Superenhancer to gene correlations and distances. (XLSX)

S5 Table Groups specific putative superenhancer associated genes. (XLSX)

S6 Table Putative tumor-specific SE driven genes for CRISPR screen. (XLSX)

S7 Table CRISPR dropout screen results. (XLSX)

S8 Table CRISPR gRNA library sequences. (XLSX)

S9 Table BAMBI-distal superenhancers correlating with dependency. (XLSX)
