# Supplementary figures and images for: Super-enhancer acquisition drives oncogene expression in triple negative breast cancer
Source: PLoS One. 2020 Jun 25;15(6):e0235343. doi: 10.1371/journal.pone.0235343 (PMC7316302; doi:10.1371/journal.pone.0235343)

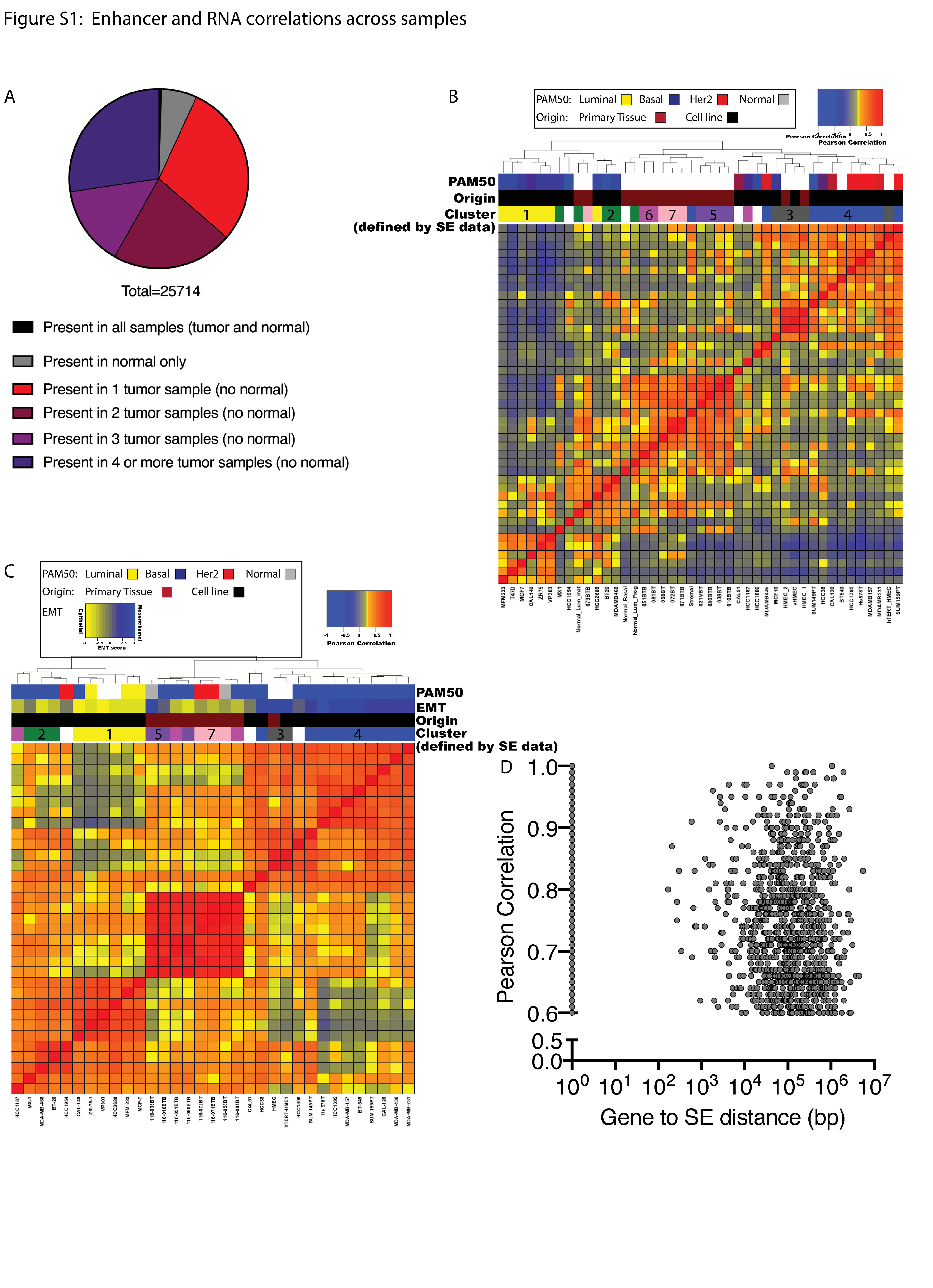

Supplement: S1 Fig — A) Pie chart shows the distribution of enhancers that fall into the indicated categories. B) Hierarchical clustering heatmap of Pearson correlations of top 10% most variable enhancers across all samples. C) Hierarchical clustering heatmap of Pearson correlations using RNA-seq values of top 10% most variable genes. D) Scatterplot shows the distance of the best correlating genes to their associated SE. (TIF) [file pone.0235343.s001.tif]

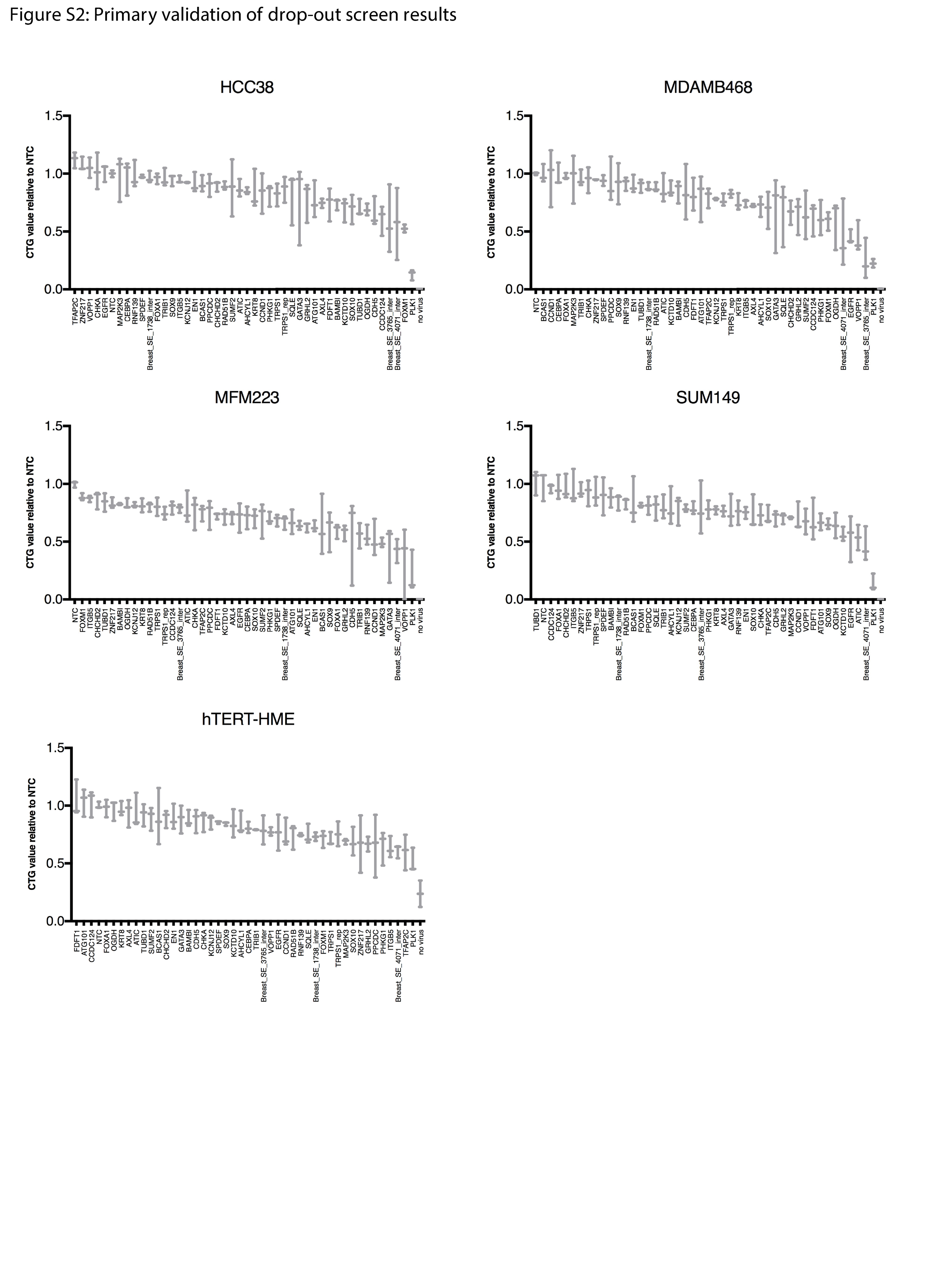

Supplement: S2 Fig — Plots show arrayed validation of primary screen hits for all CAS9 lines. Whiskers denote max/min values. Middle bar indicates mean value. (TIF) [file pone.0235343.s002.tif]

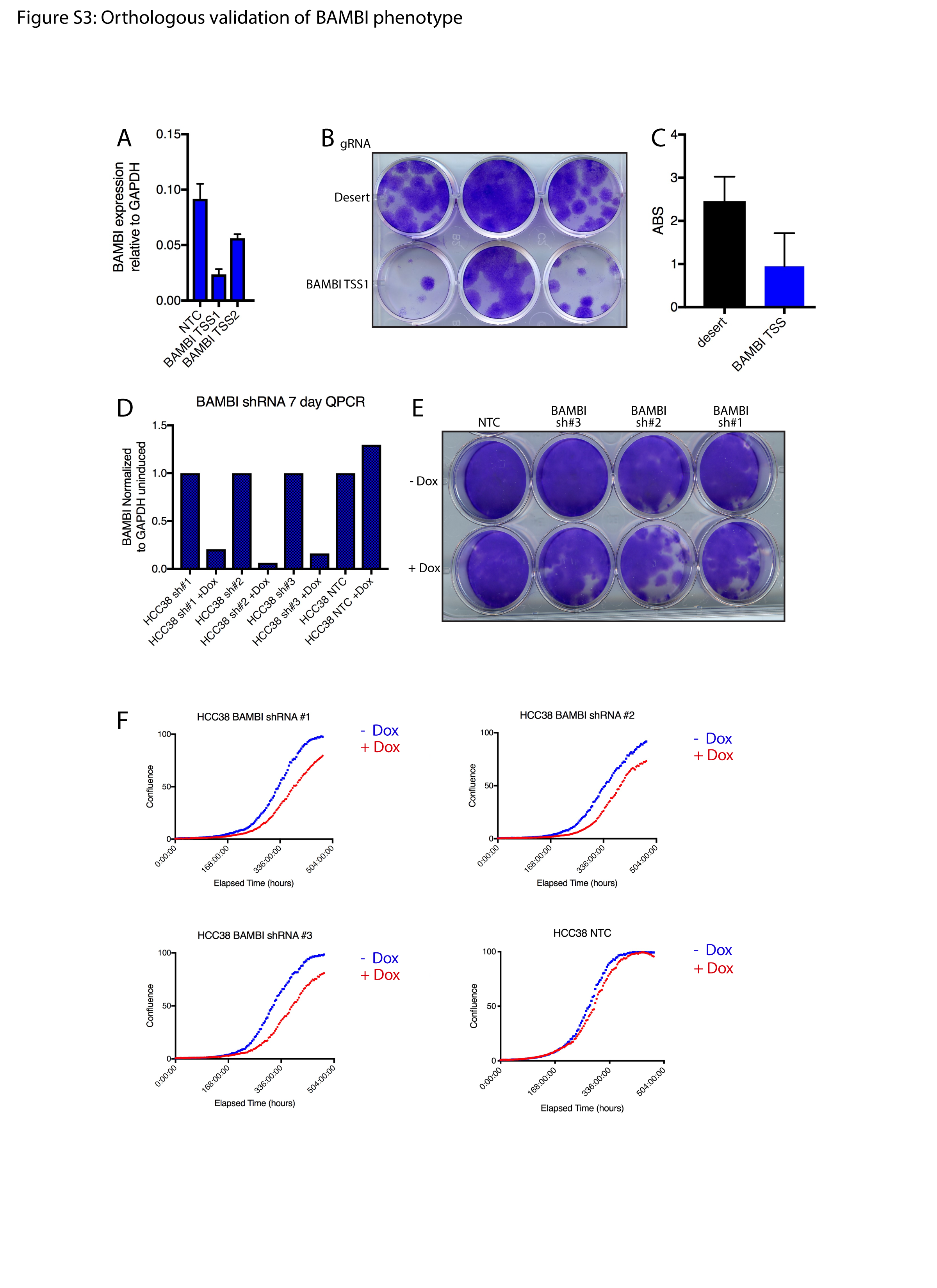

Supplement: S3 Fig — A) RT-qPCR quantification of BAMBI TSS-targeting dCAS9-KRAB guides in HCC38 cells. B) Clonogenic assays for BAMBI-TSS and “gene desert” targeting gRNAs in dCAS9-KRAB HCC38 cells. C) Quantification of clonogenic assay wells. D) RT-qPCR quantification of shRNA knockdown of BAMBI upon doxycycline induction for 3 independent shRNA constructs, and a non-targeting control shRNA at 7 days post-induction. E) Clonogenic assays of HCC38 cells after doxycycline induction of the indicated shRNAs. F) Incucyte growth assays of HCC38 cells after doxycycline induction of the indicated shRNAs. (TIF) [file pone.0235343.s003.tif]

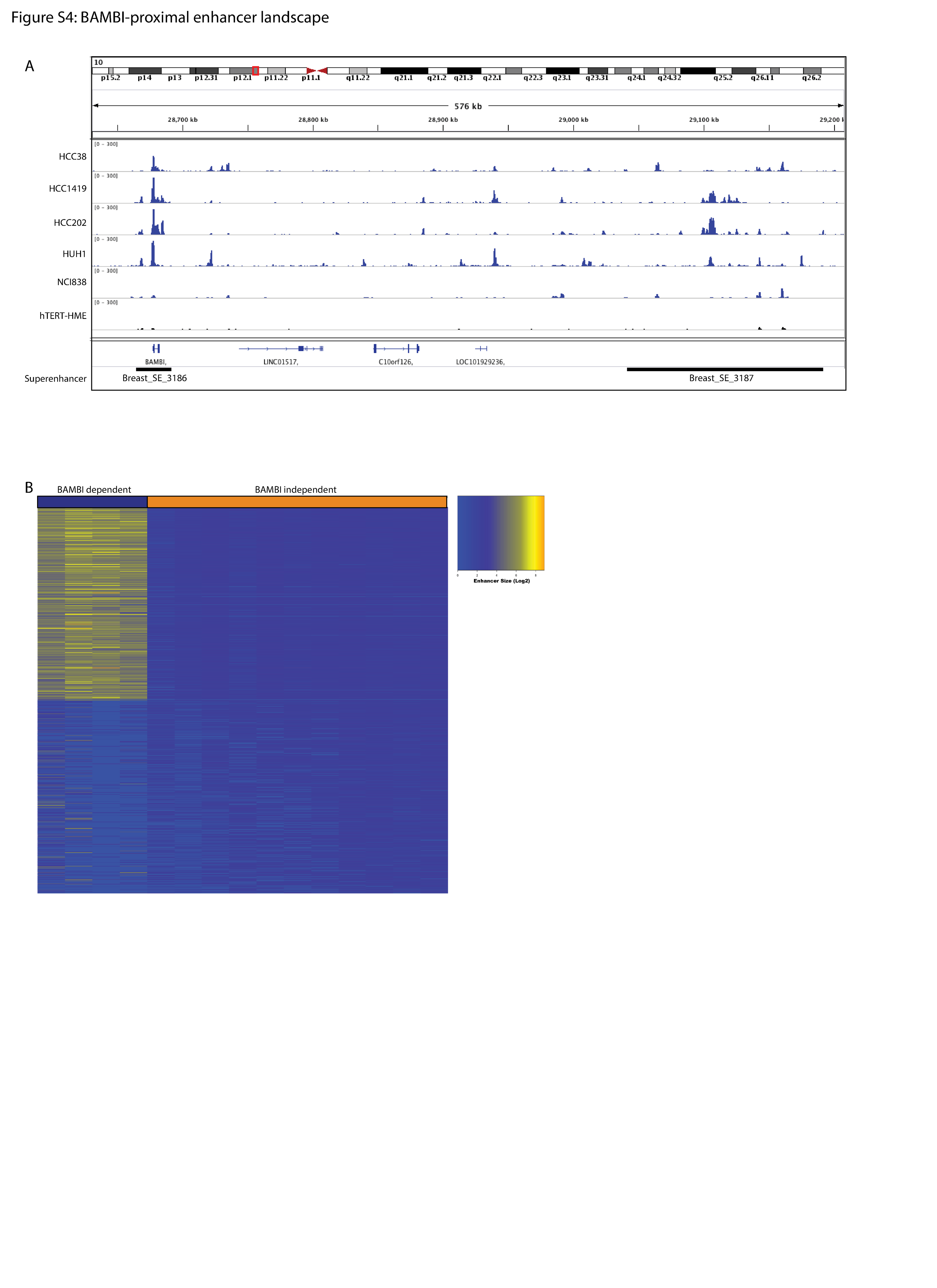

Supplement: S4 Fig — A) H3K27ac ChIP-Seq tracks of the BAMBI-adjacent region for the indicated cell lines. BAMBI associated superenhancers are indicated below the tracks. B) Heatmap showing top correlating SEs with BAMBI dependence. (TIF) [file pone.0235343.s004.tif]

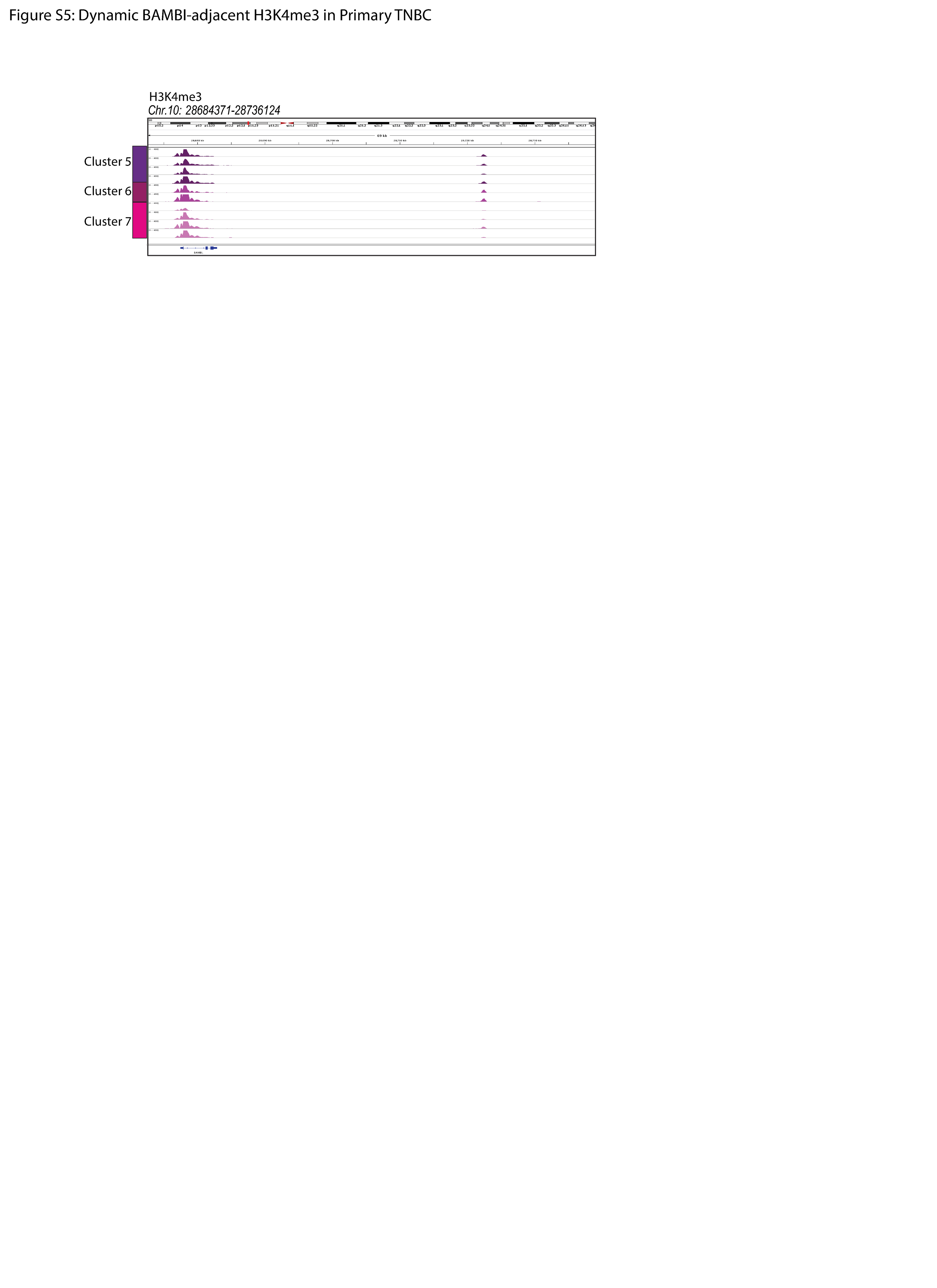

Supplement: S5 Fig — Tracks show H3K4me3 signal at the BAMBI locus in primary TNBC samples. (TIF) [file pone.0235343.s005.tif]
